# Organ Chips and Translational Research: Identifying and Examining New Ethical Issues

**DOI:** 10.1002/eahr.70031

**Published:** 2026-07-02

**Authors:** Melanie Jeske

**Affiliations:** ^1^ Assistant professor in the Center for Medical Ethics and Health Policy at Baylor College of Medicine

**Keywords:** new approach methodologies (NAMs), organ‐on‐a‐chip technologies, induced pluripotent stem cells (iPSCs), human donor data, human‐based research technologies, animal models, conflicts of interest, FDA Modernization Act 2.0

## Abstract

Organ chips, also known as organ‐on‐a‐chip devices, tissue chips, or microphysiological systems, have emerged over the last decade as a promising translational technology amidst growing concern about the translational crisis between laboratory research and patient bedside. Pointing to high rates of failure between nonhuman animal models and safety and efficacy in humans, organ chips and similar new approach methods have attracted substantial public and private investment. As human‐cell‐based alternatives to animal models, organ chips promise more predictive, efficient, and ethical platforms for pharmaceutical and toxicity testing. Engineered cultivation systems that enable cells to assemble into tissue‐like structures (e.g. kidney, brain, liver), organ chips mimic tissue architecture and function and live for extended periods of time. This essay considers the translational bioethics issues raised by organ chips, including those that arise early in development such as the obfuscation of cell‐origin data, representation in design, and the normalization of conflicts of interest within commercialization‐oriented translational science efforts. Integrating a translational bioethics lens from the outset, rather than deferring social and ethical implications analysis to downstream points in technology development and adoption, is essential to realizing the translational promise of organ chips while avoiding the reproduction of existing inequities and ethical conundrums.

Nonhuman animal models have been foundational to biomedical advances over the last century.^1^, ^2^, ^3^ Much of what we know about how humans metabolize drugs and how drugs work is first established in animal models. Since 1938, the Federal Food, Drug, and Cosmetic Act has mandated the use of animal models in pharmaceutical safety and efficacy testing to ensure there is robust evidence before moving to in‐human clinical trials. However, there are well‐known translational problems with animal models, namely the high rate of failure to predict both toxicity and efficacy of drugs in humans, which is often cited at over 90%.^4^ In recent years, many strides have been taken to reduce translational failures by introducing human‐based models into the earliest stages of pharmaceutical testing. These novel technologies introduce important questions for translational bioethics.

The FDA Modernization Act 2.0 was passed in 2022 to help address translational challenges associated with animal models. This legislation struck down the animal testing mandate, allowing drugs to move to clinical trials without animal testing data.^5^ It encouraged the uptake of new approach methodologies (NAMs) that are human‐based modeling technologies—such as organ‐on‐a‐chip technologies, organoids, and computational technologies like digital twins—technologies that promise better predictive capacities than nonhuman animal models. In April 2025, the US Food and Drug Administration (FDA) underscored this commitment, releasing a roadmap for how to achieve the reduction of animal testing in preclinical studies, stating that their 3‐5 year goal is to “make animal studies the exception rather than the norm.”^6^

Later that month, the National Institutes of Health (NIH) launched its own initiative to prioritize human‐based research technologies.^7^ In July, the NIH announced that it would no longer issue notice of funding opportunities exclusively supporting animal models and would limit or specify the types of models that must be used.^8^ By bringing in “the human” earlier in the testing process through the use of NAMs, the hope is that translational failure rates will decrease.

As a sociologist interested in how biomedical innovations emerge, are adopted, and change scientific practices, I have been analyzing the emergence and uptake of NAMs, specifically organ‐on‐a‐chip technology (hereafter organ chips), over the last decade. In this time, I have watched the hype around organ chips grow, as they progressed from a technology at the margins of biomedical research to becoming more mainstream as researchers have worked to make these models more accurate, valid, and reliable. I have witnessed how organ chips have been framed as “the future of medicine,”^9^ and the key to saying “goodbye to animal models.”^10^ Indeed, organ chip hype has often overlooked how organ chips raise new and enduring social and ethical questions, with hype focusing on their productive potential to replace animals in pharmaceutical testing. In this essay, I raise some of these bioethics issues, particularly around the sourcing of human cells and researchers’ potential conflicts of interest.

## The Hope and Hype of Organ Chips

Organ chips are fundamentally a translational technology that emerged in the context of growing concerns about translational lags and “the valley of death” between laboratory bench and patient bedsides that marked investment in translational science and medicine in the mid‐2000s.^11^, ^12^, ^13^ In 2010, a team of researchers published a paper in *Science* reporting on their creation of a “chip” platform that modeled human, organ‐level lung function.^14^ They claimed this technology, because it used human cells, provided a more accurate way to model the human body and its responses to pharmaceuticals and chemical exposures than animal models. The researchers predicted that this organ chip technology could one day replace animal models in pharmaceutical and toxicity research by introducing human‐based models in the earliest stages of safety and efficacy testing, thereby making these stages more efficient and cheaper. For a modern biomedical research enterprise built on animal models, this was a bold proposition, and one that continues to live in a space of potentiality: such a change relies not only on developing effective, reliable alternatives to animal models, but also requires changes to longstanding, deeply entrenched scientific norms.

Organ chips became a key area of investment when the NIH created the National Center for Advancing Translational Sciences (NCATS) in 2012. NCATS's investment in the development of organ chips continues to date. NCATS's Tissue Chip Program has funded the development of a wide range of organ chip systems and specific disease models, and has centralized efforts to validate and scale these models. For example, NCATS recently sent organ chips to the International Space Station to study aging and fielded clinical trials on chips.^15^ In the fifteen years since that original publication in *Science*, public and private investment in organ chips has vastly grown, and researchers across the world have taken to developing organ chips. In 2016, organ chips made the World Economic Forum's list of top 10 emerging technologies.^16^ Scientific publications on organ chips have skyrocketed from one publication in 2010 to over two thousand publications in 2025. Sociologists of science might suggest that organ chips have become something of a scientific bandwagon.^17^

**Figure 1:**
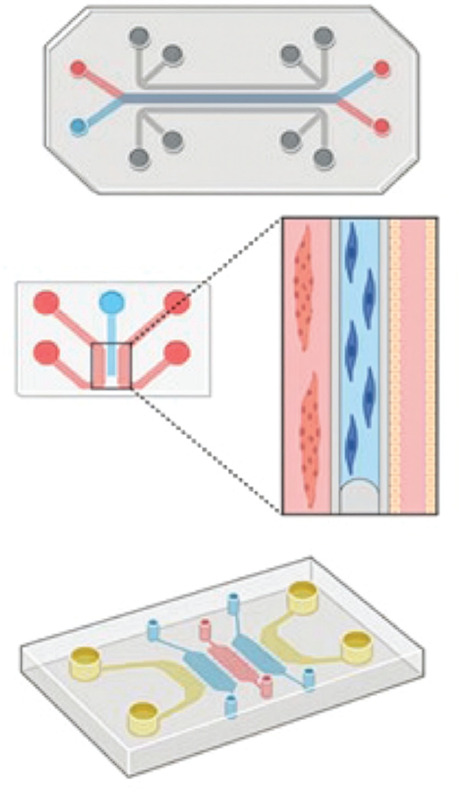
Renderings of Organ Chips

But what exactly are organ chips and what makes them so promising? Organ chips, often called organ‐on‐a‐chip, tissue chips, or microphysiological systems, are engineered cultivation systems that enable human cells to assemble into tissue‐like structures (e.g., kidney, brain, gut) that mimic tissue architecture and function. Cell cultures live on the chip for extended periods of time and are matured to the specialized cultures of the minimally functioning units of organs of interest. Chip platforms are typically made from a clear polymer with tiny channels and reservoirs etched into it. The chip design enables control of microtissue structure and allows researchers to model microfluidic flows and physical stimuli, such as electrical stimulation and mechanical forces. For instance, a lung chip is made to “breathe” with vacuum channels and an eye chip “blinks” with a sliding component. Chips also enable 3D models and can integrate co‐cultures of other tissue types into a model, and emulate *in vivo* environments (see figure 1).^18^

Different human cells can be used in these models, and most are created using induced pluripotent stem cells (iPSCs), commercially purchased or derived from primary donor cells. IPSCs derive from adult skin or blood cells that have been “reprogrammed” back to an embryonic‐like state. From there, cells can be programmed to become specialized cells characteristic of a specific organ that is of interest. Chemical compounds or pathogens can then be administered to gauge the reaction of human cells, offering insight into predicted human response.

Organ chips offer the ability to test chemical compounds on actual human cells—and to observe effects of a drug throughout multiple organs—without harm to human or animal life: the only potential death is to cells. Organ chips promise to reduce waste in terms of animal life as well as economic waste of time and resources spent on testing compounds that would have gone on to fail in humans. They promise to offer insight into human physiology in areas that have remained off limits or have been neglected. And they also promise to unlock potential compounds that could have gone on to be safe and effective in humans but failed in preclinical studies. Examples include the oft‐cited case of aspirin,^19^ which proponents of NAMs point to as evidence of an impactful drug that never would have made it to market if it had been required to be tested in animals because it is toxic to them.

Organ chips, and NAMs more broadly, are translational models that stand to solve these pressing problems. However, models create connections between different kinds of things: species to species, virtual to physical, hypothetical to actualized. They always require expertise, iteration, and interpretation to make sense of the data they provide.^20^, ^21^ They require expertise to understand how a model—which always changes, manipulates, and renders something simpler—can be translated to the thing the model represents. In other

**Moments when new scientific practices, norms, and technologies are not yet established present a window into thinking more expansively about their social and ethical implications while they are still being shaped and before they may cause harm.**

words, the work of translating between nonhuman and human may be eliminated with organ chips, but the work of translation between a model and the human it is meant to provide insight into remains. Analyzing how this work happens and how organ chips are constructed is essential to understanding and anticipating the ethical and social concerns they may raise. This is where science and technology studies (STS) approaches are particularly important: it is in and through these interpretations and translations that potential ethical concerns arise, particularly around representation, the erasure of human donor data, and how researcher conflicts of interest may shape design decisions. For translational bioethics, then, ensuring that bioethicists have the right research methods to observe these decisions and ask experts about them is essential.

## Organ Chips Raise Important Ethical Questions

Organ chips intersect with longstanding ethical challenges in pharmaceutical testing and raise new and enduring social and ethical questions. Importantly, they enable testing that would otherwise be regarded as unethical, like biological warfare agents and countermeasures.^22^ They open up areas that have been neglected, at least in part, because of ethical concerns, such as models of the female reproductive conditions—which are challenging to study in animal models because of differences in reproductive systems—and the fetal‐maternal interface and drug metabolism during pregnancy.^23^ Because organ chips exist in the domain of cellular technologies, ethical concerns have largely been constricted to issues regarding consent from humans who donate their cells for research, particularly when primary cells from patients are used to create iPSCs. Generally speaking, these concerns can be grouped with those bioethics researchers have raised about organoids, many of whom have focused on brain organoids specifically.^18^ Yet, there are other important social and ethical issues, specifically around equity and stratification in how models are built and who may benefit downstream, and researcher conflicts of interest that have been normalized in translational science and medicine.

While bioethicists often worry about issues of equity and stratification once new biomedical technologies and therapeutics are being implemented in clinical care and research practice, the seeds of downstream stratified benefit are planted early on in their development. Which humans get modeled? What cells lines derived from iPSCs become standard? In organ chip publications, cell data is often omitted even in cases where it is collected. Researchers suggest this is, in part, because the publications are often focused on the model itself—proof of concept that, for instance, specific kidney or cardiovascular tissue function can be replicated on the chip platform. Yet, the origins of cells—and omission thereof—may come to matter, as these models are not only used to screen drugs, but also used to probe into how tissues and organs function and how disease forms and progresses. What and who is represented on those chips, even in proof‐of‐concept stages, should be made clear, and be properly tracked throughout their development and clearly stated in publication.

Ample scholarship on inclusion and exclusion in biomedical research forecasts this concern.^25^, ^26^, ^27^, ^28^, ^29^ The promissory vision of organ chips *eventually* being made, but not quite yet, for particular groups or other personalization (“you‐on‐a‐chip”), truncates these concerns by deferring them to a later date. Why wait, given that these technological innovations are implemented in an existing, well‐understood climate of health care inequity? Access to cutting‐edge biomedical technologies and interventions are stratified, yet interventions toward equity could begin at the start if this were an essential element in biomedical research.

Organ chips are celebrated for their translational potential. Getting therapies and new technologies to patients is a key goal of translational medicine, and that has been accomplished through public‐private partnerships, encouraging academic researchers to pursue the commercial potential of their research, often by launching start‐ups and creating relationships with potential investors who are willing to fund the risky stages of research and development with the promise of future returns.^30^, ^31^ Since the 2003 NIH Roadmap, which outlined numerous translational research efforts, enhanced relations between government, researchers, and industry have been understood to be essential to successful translation.^11^, ^12^ While translational research aligns these interests, some types of these arrangements have historically been understood as creating potential conflicts of interest.

Simply put, there are ethical concerns about conflicts of interest that have been normalized in translational research. Commercial or privatized interests should not be given priority by shaping research objectives in ways that favor commercial over public benefit.^31^ Biomedical research should focus on understanding and ethically addressing how the goals of commercialization and attracting investors create conditions under which researchers might feel pressure to inflate their successes. For example, in these pressurized arrangements, researchers may contend that technologies are more promising, more useful, than they are. This overpromising and underdelivering—or failing altogether—has most notably occurred in the hype‐filled Silicon Valley biotech space, including specific cases of deceit.^32^ As these activities increasingly become normalized parts of doing academic biomedical research, ethical reflection and practice is essential. Ethical practice in biotechnology development must include thoughtful engagement that acknowledges the decades of evidence that shows how conflicts of interest influence research practice and its results.^33^, ^34^ Transparency around conflicts of interest has become standard, but these are often broad disclosures that simply state the nature of a conflict (e.g., that an author received funding from a particular entity). We could consider requiring statements about how goals of scalability and commercialization shape technological design decisions.

## Integrating Social and Ethical Concerns from the Outset of Research

Sociologists and historians of science and medicine have shown time and again that science is slow to change. What appear as paradigm shifts are often not; rather, they are a new configuration of practices that have been in the works for some time. Such reorganization has the potential for change, but it is not often transformational. The development of technologies like organ chips and the passage of the FDA Modernization Act 2.0 (and potentially 3.0) has been heralded as a sea change, but whether and how lasting changes will be made remains to be seen. Animal models will continue to be essential in biomedical research—from nematodes, to rodents, to dogs, to primates. They teach us things about our bodies, they teach us things about their bodies, and they teach us things that cannot be engineered on a chip. Even organ chip researchers agree, noting the foundational role of animal models in biomedicine.

More broadly, many researchers defend the use of animal models; after all, so many advances have been made possible using them.^35^ And perhaps most importantly, in order for organ chips and NAMs to be most beneficial and to avoid harm—alongside animal models or instead of them—they must be rigorously validated.^36^ Even then, perhaps it is best to think about NAMs as supplemental models rather than replacements. However, recent legislative wins and institutional priorities suggest that this is a moment when things are shifting in favor of NAMs, particularly in the pharmaceutical testing space. Indeed, in September 2025, three years after the FDA Modernization Act 2.0 passed, the biotech company Qureator announced that their vascularized tumor immune microenvironment model supported the first approved investigational new drug application using data generated entirely from their organ chip technology.^37^

## Embedding Social and Ethical Analysis Throughout the Life Course of Novel Technologies

Back when organ chips were just beginning to get mainstream media coverage, an episode of National Public Radio's *All Things Considered* featured a segment where they discussed the latest advances and potential of these technologies. The host of the show included a brief discussion of ethics, noting that “the researchers stress that they only want to use these models to study anatomy and come up with new treatments.”^38^ They asked for input from a bioethicist, who suggested that while researchers creating organ chips may have “good intentions,” because the research was advancing at “such a rapid pace,” researchers would not be able to control how others took up the technologies and toward what ends they used them. It was at that juncture, the ethicist suggested, that ethical concerns arose. Potential ethical conundrums and social implications were being circumscribed: it was not a question of how the developers of the technologies themselves could fall prey to ethical missteps or how these novel technologies might entrench forms of bias, but rather how *others* might misuse them. Moreover, it was presented in such a way that the very tasks at hand—creating these models to study anatomy and test new medical treatments—could not themselves be sites of ethical quandaries. To be clear, the intent here is not to question the developers of these technologies and whether they have acted or will act unethically. Rather, it is about ensuring that ethical issues will be considered from the earliest stages of research. Failing to do so likely means some extremely important issues will be overlooked, such as the omission of cell origin data and the potential for conflicts of interest.

Moments when new scientific practices, norms, and technologies are not yet established present a window into thinking more expansively about their social and ethical implications while they are still being shaped and before they may cause harm. Put simply, the less attention paid to ethical issues early on, the more opportunity there is for unforeseen and unintended ethical issues to arise. Statistician George E. P. Box famously wrote “all models are wrong, but some are useful,” a saying that many computationalists and modelers often repeat. It recognizes that all models have their limitations—that is, indeed, an inherent feature of models. But even those models that are useful ones are not necessarily free of ethical conundrums, nor are they neutral. As novel models like organ chips become used more frequently in biomedical research, it is important to anticipate (and mitigate where possible) the social and ethical questions they raise. Doing so requires that those with expertise in STS and bioethics are consulted and taken seriously along the way.

## DISCLAIMER

All opinions expressed in this essay are those of the author.
